# Two Novel Polysaccharides From *Clitocybe squamulosa*: Their Isolation, Structures, and Bioactivities

**DOI:** 10.3389/fnut.2022.934769

**Published:** 2022-06-30

**Authors:** Dongdong Guo, Jiayu Lei, Lijing Xu, Yanfen Cheng, Cuiping Feng, Junlong Meng, Mingchang Chang, Xueran Geng

**Affiliations:** ^1^College of Food Science and Engineering, Shanxi Agricultural University, Taigu, China; ^2^Shanxi Key Laboratory of Edible Fungi for Loess Plateau, Taigu, China; ^3^Shanxi Engineering Research Center of Edible Fungi, Taigu, China

**Keywords:** *Clitocybe squamulosa* polysaccharides, purification, structural characterization, bioactivity, physicochemical properties

## Abstract

The crude polysaccharides from the fruiting bodies of *Clitocybe squamulosa* (CSFP) were isolated by hot-water extraction. Two novel polysaccharides, CSFP1-β and CSFP2-α, were further purified by DEAE-52 anion exchange and Sephacryl S-400 gel filtration chromatography, and the purities reached 98.44 and 97.83%, respectively. The structural characteristics and bioactivities of CSFP, CSFP1-β, and CSFP2-α were identified by the combination of chemical and instrumental analysis. Results showed that CSFP was formed by the aggregation of honeycomb spherical materials; CSFP1-β and CSFP2-α were interwoven by reticular and fibrous structures, respectively. Purified components of both CSFP1-β and CSFP2-α showed typical infrared absorption peaks of polysaccharides, and contents of nucleic acid and protein decreased significantly. Simultaneously, CSFP with a molecular weight (Mw) of 1.948 × 10^4^ Da were composed mainly of glucose, mannose, galactose, and rhamnose. CSFP1-β was composed mainly of glucose, galactose, and mannose, while CSFP2-α was composed of glucose, and both their *Mw* distributions were uneven. Compared with CSFP, the antioxidant activities of CSFP1-β and CSFP2-α were significantly improved (*p* < 0.05), and they both showed good abilities to bind free cholesterol and bile acid salts *in vitro*. The binding abilities of the two compounds were found to be 68.62 and 64.43%, and 46.66 and 45.05 mg/g, respectively. CSFP, CSFP1-β, and CSFP2-α had good bacteriostatic effects with a linear increasing relationship to increasing concentration. In addition, CSFP promoted the growth of RAW264.7 cells and has potential immunomodulatory, anti-inflammatory, and anti-tumor activities.

## Graphical Abstract

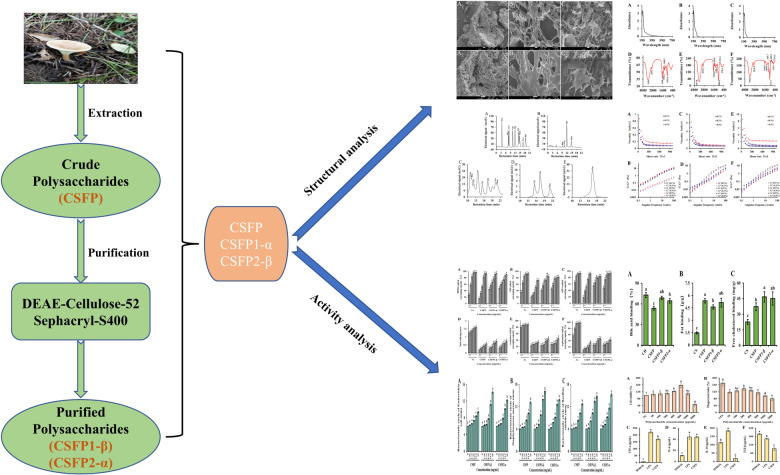

## Highlights

- *Clitocybe squamulosa* is a precious wild edible fungus, which is rich in a variety of nutrients.
- Two polysaccharides named CSFP1-α and CSFP2-β were isolated from *Clitocybe squamulosa.*
- CSFP1-α and CSFP2-β show good biological activity.
- CSFP can promote RAW264.7 cell growth.

## Introduction

Polysaccharides are a natural biomaterial, widely present in animals, plants, and microorganisms. Previous research found that polysaccharides exhibit a variety of health promotion and drug regulation effects, such as antioxidant (1), anti-tumor (2), and immune regulation (3), and also have the potential to prevent the effects of hyperglycemia and high cholesterol (4). In recent years, functional mushroom-derived polysaccharides have been widely applied in medicine, food, and other fields because of their multiple biological effects and low toxicity, and lack of severe side effects (5, 6). So far, studies have reported the *in vitro* bioactivities of a variety of natural mushroom polysaccharides. For example, a novel acid heteropolysaccharide isolated from a spent mushroom substrate of *Pleurotus eryngii* exhibited strong superoxide radical [EC 50 = (0.33 ± 0.02) mg/ml], hydroxyl radical [EC 50 = (1.19 ± 0.02) mg/ml], and DPPH radical [EC 50 = (0.52 ± 0.02) mg/ml] scavenging rates in a dose-dependent manner (7). Hao et al. (8) proved that *Pleurotus citrinopileatus* polysaccharides showed non-competitive inhibition of α-glucosidase activity (IC 50 = 0.556 mg/ml) and could reduce the oxidative stress in HepG2-IR cells. Wang et al. (9) prepared polysaccharides from *Armillariella tabescens* mycelia (PAT) with a remarkable inhibitory effect on the growth of typical food-borne pathogenic bacteria using a green technique. The results showed that PAT could inhibit the growth of *Escherichia coli*, *Proteus vulgaris*, *Bacillus subtilis*, and *Staphylococcus aureus* cells, with minimum inhibitory concentrations of 0.5, 1.0, 4.0, and 4.0 mg/ml, respectively. Moreover, Xiao et al. (10) extracted and purified a rare wild *Lactarius deliciosus* fruiting body polysaccharides (LDP-1). LDP-1 with a *Mw* of 9.8 × 10^5^ Da showed immunological activity against RAW264.7 cells, which can promote the proliferation and phagocytosis of RWA264.7. The cytokines of TNF-α, IL-1β, and IL-6 were secreted in a concentration-dependent manner (at 4.83, 17.8, and 11 times that of the control, respectively). Therefore, mushroom polysaccharides with natural activity have very important research value.

In addition, the bioactivity and structural characteristics of mushroom polysaccharides have been explored using various analytical methods, which found that there was a close relationship between them (11). Ji et al. (12) discovered that ginseng (*Panax ginseng C. A. Meyer*) polysaccharides rich in uronic acid often have higher biological effects, and the α-(1→4)-GalpA and α-(1→4)-Galp components in the main chain are essential for their anti-inflammatory and antiviral effects. Li et al. (13) implied that *Hericium erinaceus* polysaccharide (HEP) with immune activity usually had a relatively large *Mw* and that with low *Mw* had strong antioxidant activity. The activity of HEP was significantly correlated with *Mw*. Moreover, more electron-withdrawing groups such as carboxyl or carbonyl groups in a polysaccharide could lower the dissociation energy of the O-H bond, thus resulting in the greater release of hydrogen atoms. Chen et al. (14) purified a homogeneous *Lentinus edodes* polysaccharide (LEPA1), which contains no uronic acid and exhibited weak scavenging activity on superoxide radicals, as compared with other polysaccharide fractions containing uronic acid and ascorbic acid. Therefore, it is important to develop new polysaccharide resources from mushrooms and explore their structure–activity relationship in practical production and application.

*Clitocybe squamulosa* is a precious wild edible mushroom, which is widely distributed in Wutai Mountain Ecological Park, Shanxi Province, China. Its fruiting bodies have a firm and tender texture, unique flavor, and are rich in nutrients, making them popular among consumers (15). Due to the differences in purity and structure–activity relationships of polysaccharides, mushroom-derived polysaccharides from different sources impart different physiological functions. So far, most of the research on *C. squamulosa* polysaccharides (CSFP) has focused on the extraction and utilization of crude polysaccharides, which is not conducive to the in-depth exploration of the structure–activity relationship of polysaccharides. There have been no reports until now concerning the isolation, purification, and structural elucidation of CSFP. The main purpose of this study is to determine the processing and structural characteristics of high-purity polysaccharides isolated from *C. squamulosa* and evaluate their potential biological activities *in vitro*. The research may provide a theoretical basis for further understanding of the structure–activity relationship of polysaccharides.

## Materials and Methods

### Materials and Chemical Reagents

The *Clitocybe squamulosa* were provided by the Edible Fungi Center of Shanxi Agricultural University (Shanxi Province, China). Mouse monocyte macrophage RAW264.7 cells were purchased from Shanghai Zhongqiao Xinzhou Biotechnology Co., Ltd (Shanghai Province, China). DEAE-cellulose-52 and Sephacryl S-400 were purchased from Solarbio (Beijing, China) and GE Healthcare (Chicago, IL, United States). All other chemical reagents were of analytical grade.

### Extraction and Purification of CSFP

The CSFP could be extracted according to a previously reported method (16). In short, the *C. squamulosa* fruiting body powder was mixed with ultrapure water in a ratio of 1:30 (*w/v*). Then, the mixture was filtered after 3.6 h in a water bath at 80°C and concentrated with a rotary evaporator (IKA, China). The zinc acetate potassium ferrocyanide method was adopted for protein removal, followed by ultrapure water dialysis (molecular retention 3,500 Da). The dialysate and absolute ethanol were alcohol-precipitated overnight in the ratio of 1:4, and the sediment was collected, freeze-dried, and stored at low temperature for later assays. CSFP could be further isolated and purified according to the previously reported method (2). Briefly, CSFP could be fully dissolved in ultrapure water, configured into an aqueous solution (10 mg/ml), loaded into a DEAE-cellulose-52 ion exchange column (2.8 cm × 32 cm), and then eluted successively with 0, 0.1, and 0.3 mol/L NaCl solution (flow rate: 4 ml/min). The eluted components were collected and reloaded into a gel permeation chromatography column (Sephacryl S-400 (XK26 × 100 cm)), and then eluted with 0.2 mol/L NH_4_HCO_3_ solution at a flow rate of 3 ml/min. The content of polysaccharides in the collecting tube was determined by a phenol-sulfuric acid method (17), and the elution curve was drawn according to OD_490_ data. The main fraction was collected for subsequent analyses.

### Chemical Composition

The method of determination of polysaccharide content was consistent with that described in section “Extraction and Purification of CSFP,” and glucose was used as the standard. The reducing sugar (*C*_*R*_) content was determined by the DNS method with glucose as a standard (18). The content of uronic acids was determined by the sulfuric acid-carbazole method with galacturonic acid as a standard (18). The protein content was determined by Coomassie brilliant blue method with BSA as a standard (19).

### Structural Analysis

#### Scanning Electron Microscopy

Three different polysaccharide samples (CSFP, CSFP1-β, and CSFP2-α) were coated on the sample sticking table, respectively, and gold-spray-coated. Scanning Electron Microscopy (SEM) with a 20.0-kV accelerating voltage (Zeiss Merlin, Germany) was adopted to observe the morphological characteristics of the samples at different magnifications (2).

#### Ultraviolet–Visible Absorption Spectra

Freeze-dried samples (CSFP, CSFP1-β, and CSFP2-α) were prepared into 1 mg/ml solutions with ultrapure water, respectively. The absorption peaks of polysaccharide samples at 260 and 280 nm were measured using a Cary60 UV spectrophotometer (Agilent, United States) in the range of 190–800 nm (20).

#### Fourier-Transformed Infrared Spectroscopy

The powdered samples (CSFP, CSFP1-β, and CSFP2-α, respectively) weighing 1 mg and dry KBr powder (150 mg) were mixed evenly, pressed in an FW-4A powder tablet press, and the pressed samples were analyzed using a spectrophotometer (TENSOR 27, Bruker, Germany) in the range of 400–4,000 cm^–1^.

#### Monosaccharide Composition

The monosaccharide composition of different samples (CSFP, CSFP1-β, and CSFP2-α) was measured by using ICS5000 + ion chromatography (Thermo Scientific, United States). The sample loading quantity was 20 μl, and the chromatographic conditions included mobile phase A (ultrapure water), mobile phase B (100 mmol/L NaOH solution), and column temperature (30°C) (21).

#### Molecular Weight Distribution

The *Mw* of different samples (CSFP, CSFP1-β, and CSFP2-α) was measured by high-performance gel permeation chromatography (HPGPC) with 18-angle laser light scattering (Wyatt Technology, United States). We eluted 0.1 mol/L NaNO_3_ (at a flow rate of 0.5 ml/min), and then injected 20 μl samples (5.0 mg/ml) at 35°C (22).

#### Rheological Properties

The effects of CSFP, CSFP1-β, and CSFP2-α samples at different concentrations (1, 3, and 5%) on the viscosity of polysaccharide solution were measured using MCR 102 rheometer (loaded with lamina CP 50-1, diameter 50 mm, Anton Paar, Graz, Austria). Similarly, the variations in storage modulus (*G*’) and loss modulus (*G*”) of 1, 3, and 5% polysaccharide solutions at oscillation frequencies from 0.1 to 100 rad/s were also studied (23).

### *In vitro* Bioactivity Analysis

#### Antioxidant Activities

The DPPH, ⋅OH, and ABTS radical scavenging abilities of different samples (CSFP, CSFP1-β, and CSFP2-α) were determined strictly based on the methods of a correlation test kit (Solarbio Science, Beijing, China). The total reducing power of different samples could be determined according to the previously reported method (24, 25). In short, we added 1 ml of polysaccharide solution, 2.5 ml of phosphoric acid buffer (pH 6.6, 0.2 mol/L), and 2.5 ml of 1% potassium ferricyanide solution to the test tube, mixed these evenly, and placed the specimen in a water bath at 50°C for 20 min. We then added 2.5 ml of 10% trichloroacetic acid solution and centrifuged the specimen at 1,000 rpm for 10 min. We mixed 2.5 ml of supernatant, 2.5 ml of distilled water, and 6.5 ml of 0.1% ferric chloride solution evenly, stood it for 10 min, and measured the absorbance at 700 nm. The reducing power was represented by the absorbance value. Vitamin C (Vc) was used as a positive control.

#### Enzymatic Inhibitory Activities

The α-glucosidase and α-amylase inhibitory activities of different samples (CSFP, CSFP1-β, and CSFP2-α) were assessed according to the previously reported methods (26, 27). Acarbose (Ac) was used as a positive control.

#### *In vitro* Binding Properties

The binding capacities (bile acid, fat, and free cholesterol) of different samples (CSFP, CSFP1-β, and CSFP2-α) *in vitro* were estimated according to the previously reported methods (28). Carboxymethylcellulose sodium (CS) and cholestyramine (CH) were used as positive controls, respectively.

In short, the method for the determination of fat-binding capacity is as follows: we fully dissolved 0.5 g of polysaccharide in 10 ml of ultrapure water, adjusted the pH of the solution to 2.0 with 0.1 mol/L HCl, added 10 g soybean oil, mixed well, incubated at 37°C for 2 h, removed it, adjusted the pH to 7.6 with 0.1 mol/L NaCl solution, incubated at 37°C for 2 h, and then centrifuged it at 4,000 rpm for 20 min. Then carefully removed the unbound oil from the upper layer and weighed it, and the total amount of oil minus the amount of unbound oil is the weight of the combined fat in the sample (expressed in grams per gram of polysaccharide bound soybean oil (g/g)).

The method for determining the binding capacity of polysaccharides to cholesterol includes the following steps. Preparation of micelle solution: 1 ml of micelle solution contained 100 mmo1/L sodium taurocholate, 20 mmol/L cholesterol, 50 mmol/L oleic acid, 1,320 mmol/L sodium chloride, and 150 mmol/L phosphate buffer (pH 7.4). This solution was combined with the polysaccharide in a micellar solution, and the mixed solution was incubated at 37°C for 2 h and centrifuged at 10,000 rpm for 20 min. The supernatant was collected and the cholesterol content was determined using a kit (Solarbio Science, Beijing, China), which was expressed as the amount of bound cholesterol in milligrams per gram of polysaccharide bound cholesterol (mg/g).

The method for determining the binding capacity of polysaccharides to cholate is as follows. We took a certain amount of polysaccharide sample and mixed it with 5 ml of 0.1 mol/L hydrochloric acid solution. Afterward, the mixture was incubated at 37°C for 2 h, the pH of the mixture was adjusted to 7.6 with 0.1 mol/L sodium hydroxide solution, 10 ml of 0.1 mol/L phosphate buffer (containing 0.5 mg/ml cholate) was added, and the solution was incubated at 37°C for 2 h. The content of cholate in the supernatant was analyzed. The amount of polysaccharide bound bile salt was calculated from the difference between the total amount of bile acid salt and the amount of unbound bile salt (expressed in milligrams per gram of polysaccharide bound bile salt (mg/g)).

#### Bacteriostatic Activity

The antibacterial activities were studied against three bacterial species (*E. coli*, *S. aureus*, and *B. subtilis*) from the Engineering Technology Research Center of Edible Fungi (Shanxi, China) (following a published method (29), with slight modification). Using the K-B filter paper method, CSFP, CSFP1-β, and CSFP2-α solutions with different concentrations (0, 1.56, 3.12, 6.25, 12.5, and 25 mg/ml) were prepared, respectively, and 6-mm filter paper pieces were soaked in the prepared polysaccharide solutions. We then absorbed 100 μl of the activated strain suspension and evenly applied it on a plate prepared with LB medium. The filter paper pieces with the different polysaccharide concentrations were placed on the plate coated with a bacterial solution at equal intervals, and each group of tests could be repeated three times. The filter paper pieces soaked with sterile water were used as blank control. The plate was placed in a constant temperature incubator suitable for strain growth for 48 h, and the diameter of the bacteriostatic circle was recorded.

#### Effect of CSFP on the Growth of RAW264.7 Cells

##### Cell Culturing

Converted macrophage RAW264.7 cells were placed in the complete medium containing 1% double antibody and cultured in the incubator at 37°C and 5% CO_2_. CSFP solutions with different concentration gradients were prepared, filtered using a 0.22-um aqueous phase filter membrane, and retained for later assay.

##### Determination of Cell Viability and Phagocytic Index

The MTT method (2) was used to detect cell viability as follows: the RAW264.7 cell concentration in the logarithmic growth period was adjusted to 1 × 10^6^ pieces/ml. We added 150 μl of sample solution to a cell plate for culture over 12 h at 37°C and 5% CO_2_, discarded the medium, and added 150 μl of DMEM complete culture medium containing different concentrations of CSFP. The specimens were cultured for 24 h. Afterward, discarded the supernatant, added 10 μl of 5 mg/ml MTT solution to each well of the plate, cultured them for 4 h at 37°C, discarded the supernatant, and then added 150 μl of DMSO to each well. A normal control group and a blank control group were established at the same time. The absorbance was measured at 490 nm by a microplate reader (SpectraMax i3X, Shanghai, China).

The neutral red method (30) was used to detect cell phagocytic activity. The culture of RAW264.7 cells and the preparation of CSFP solution were the same as in the MTT method. After adding different concentrations of CSFP solution and incubating for 24 h, we removed the supernatant, added 100 μl of 0.075% neutral red PBS solution, cultured for 2 h, discarded the supernatant, washed it three times with PBS, added 100 μl of cell lysate to each well, and allowed the specimens to stand at 4°C for 12 h. The absorbance was measured at 540 nm by a microplate reader (using LPS as the positive control).

##### Determination of Cytokine Content

Taking RAW264.7 cells in the logarithmic growth period, the cell concentration was adjusted to 1 × 10^6^ pieces/ml. Then, 150 μl of 200 μg/ml CSFP complete medium was added to the test group, and 150 μl of complete medium was added to the blank control group and positive control group, respectively. Each group was cultured at 37°C and 5% CO_2_ for 12 h, whereafter the supernatant was removed. Then 150 μl of 1.0 μg/ml LPS DMEM high-sugar medium was added to the experimental group and positive control group, respectively. Next, 150 μl of DMEM high-sugar medium was added to the negative control group, and the determination was conducted after continuous culture for 4 h. The contents of TNF-α, IL-6, IL-10, and TGF- β in the cell culture medium were detected in strict accordance with the instructions supplied with the Enzyme-Linked Immunosorbent Assay (ELISA) Kits (Shanghai, China).

### Statistical Analysis

MS-Excel^®^ was used to plot the charts herein. All tests were repeated in triplicate. The results were presented as mean ± standard deviation (SD). Statistical significance analysis was undertaken using SPSS 20.0 software (IBM Inc., Chicago, IL, United States). Analysis of variance (ANOVA) and Duncan’s multiple range test (*p* < 0.05) were performed to evaluate differences between the samples.

## Results and Discussion

### Extraction, Purity, and Chemical Composition of CSFP, CSFP1-β, and CSFP2-α

**FIGURE 1:**
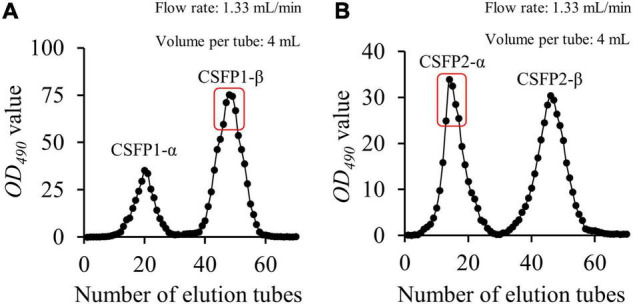
Elution profiles of CSFP in Sephacryl S-400. The sample of CSFP is loaded into the DEAE-52 anion exchange column. The collected component was named as CSFP1 after washing with ultrapure water, and then the collected component was named as CSFP2 after adding 0.1 M NaCl solution. **(A)** Elution profiles of CSFP1 in Sephacryl S-400 of the, and **(B)** Elution profiles of CSFP2 in Sephacryl S-400.

The CSFP (yield, 4.15%) could be obtained after hot-water extraction and ethanol precipitation, and further purified using a DEAE-cellulose-52 ion exchange column and a Sephacryl S-400 gel permeation chromatography column. The main fraction (Figure 1) was collected and freeze-dried to obtain the pure polysaccharide (CSFP1-β and CSFP2-α) for structural characterization (Table 1). The polysaccharide contents of CSFP, CSFP1-β, and CSFP2-α were 63.72, 98.44, and 97.83%, respectively, and the protein contents were 3.05, 1.17, and 1.19%, respectively. The results showed that purification could reduce the amounts of small molecules, such as pigments, protein, and other impurities, in crude polysaccharide samples and thereby improve the purity (2). In addition, compared with CSFP, the uronic acid contents of CSFP1-β and CSFP2-α decreased significantly (*p* < 0.05), which were 8.97 and 9.42%, respectively. It was found that the uronic acid content of natural polysaccharide may be closely related to its antioxidant activity, binding properties, and α-glucosidase inhibitory effect (30, 31). Moreover, compared with CSFP, the values of *C*_*R*_ of CSFP1-β and CSFP2-α were also significantly reduced (*p* < 0.05), but there was no significant difference between CSFP1-β and CSFP2-α.

**TABLE 1:** The content of polysaccharides, proteins, uronic acid, and reducing sugars in CSFP, CSFP1-β, and CSFP2-α.

Content (%)	Polysaccharide	Protein	Uronic acid	Reducing sugar
CSFP	63.72 ± 4.91^b^	3.05 ± 0.10^a^	11.81 ± 0.99^a^	2.60 ± 0.12^a^
CSFP_1_-β	98.44 ± 1.93^a^	1.17 ± 0.08^b^	8.97 ± 0.70^b^	1.98 ± 0.07^b^
CSFP_2_-α	97.83 ± 1.42^a^	1.19 ± 0.05^b^	9.42 ± 0.51^b^	1.95 ± 0.02^b^

*Values represent mean ± standard deviation, and different superscript lowercase letters indicate significance (p < 0.05) in each row.*

### Structural Characteristics Analysis of CSFP, CSFP1-β, and CSFP2-α

#### Scanning Electron Microscopy Analysis

**FIGURE 2:**
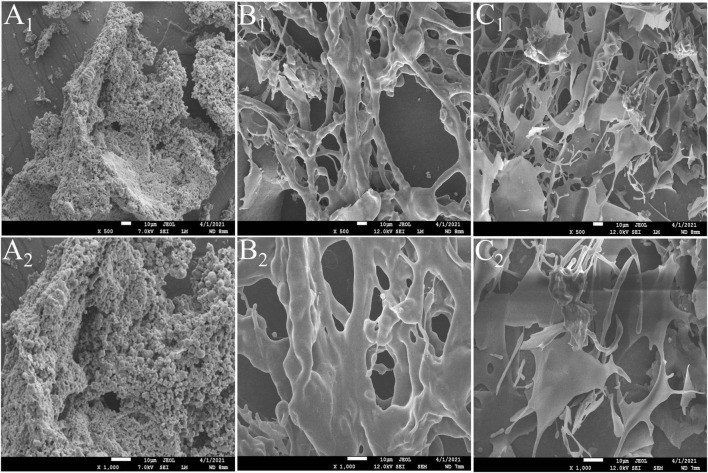
SEM images of CSFP, CSFP1-β, and CSFP2-α at ×500 and ×1,000 magnifications. **(A_1_,A_2_)** CSFP; **(B_1_,B_2_)** CSFP1-β; and **(C_1_,C_2_)** CSFP2-α.

Different micro-morphological characteristics were found to be the key factors leading to the complexity of polysaccharides (32). The microstructure of CSFP, CSFP1-β, and CSFP2-α was investigated using SEM analysis (Figure 2). The results show that CSFP could be formed by irregular spherical particle clusters in a dense honeycomb shape. After purification, there were significant differences in the microstructure between the dialyzed CSFP and the purified polysaccharide fractions (CSFP1-β and CSFP2-α). The apparent morphology of CSFP1-β was rough, and the structure remained intact and was randomly cross-linked by the network structure. However, CSFP2-α was observed to be smooth and uniform, comprising a fibrous structure similar to columnar and flake-like forms. Research showed that the surface structure of CSFP2-α was akin to that of the purified HEFP-2b component of *H. erinaceus* fruiting body polysaccharides (2). Ji et al. (33) speculated that the network structure aggregation of polysaccharide molecules may be closely related to the presence of a variety of carboxyl and hydroxyl groups. It was found that the higher content of hydroxyl and carbonyl groups in the polysaccharide chains appeared to strengthen the intermolecular and intramolecular interactions, resulting in strong molecular chain aggregation and more stable polysaccharide molecules (34).

#### Ultraviolet–Visible Absorption Spectra and Fourier-Transformed Infrared Spectroscopy Spectroscopic Analyses

**FIGURE 3:**
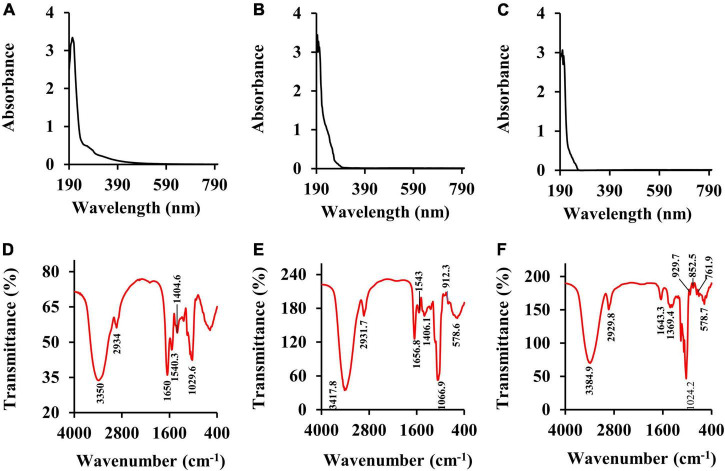
The UV spectra and FT-IR spectra of CSFP, CSFP1-β, and CSFP2-α. **(A–C)** The UV spectra, and **(D–F)** the FT-IR spectra.

The Ultraviolet-Visible absorption spectra (UV spectra) of CSFP, CSFP1-β, and CSFP2-α are shown in Figures 3A–C. No absorption peaks were observed at 260 and 280 nm, indicating that three samples contain negligible amounts of nucleic acids and proteins (35). This was consistent with the results of chemical composition wherein little protein was detected in these polysaccharide fractions (Table 1). Fourier-transformed infrared spectroscopy was mainly used to distinguish and identify some functional groups, chemical bond substituents, and pyranose ring and furanose ring structures in the natural polysaccharides (36). As shown in Figures 3D–F, briefly, the characteristic peaks of CSFP, CSFP1-β, and CSFP2-α at 3,350 cm^–1^, 3,417.8 cm^–1^, and 3,384.9 cm^–1^ were due to the tensile vibrations of O-H bonds, which displayed typical polysaccharide peaks (37). The absorption peaks at 2,934 cm^–1^, 2,931.7 cm^–1^, and 2,929.8 cm^–1^ were due to the vibrations of C-H bonds (38). The absorption bands at 1,650 cm^–1^, 1,656.8 cm^–1^, and 1,643.3 cm^–1^ were caused by the stretching vibrations of C=O and COO- bonds, indicating the presence of -COOH groups in CSFP, CSFP1-β, and CSFP2-α (39). The absorption peaks at 1,404.6 cm^–1^, 1,406.1 cm^–1^, and 1,369.4 cm^–1^ were attributed to the stretching vibration of C-O bonds, indicating the presence of -OCH_3_ groups (40). Moreover, the absorption bands at 1,043 cm^–1^, 1,066.9 cm^–1^, and 1,024.2 cm^–1^ might be a result of O-H variable-angle vibrations, indicating that CSFP, CSFP1-β, and CSFP2-α contained a pyranose ring structure (41). The absorption peaks at 912.3–929.7 cm^–1^ and 850 cm^–1^ showed that both CSFP1-β and CSFP2-α contained a β-glycosidic bond and α-glycosidic bond (19).

#### Monosaccharide Composition and Molecular Weight Analysis

**FIGURE 4:**
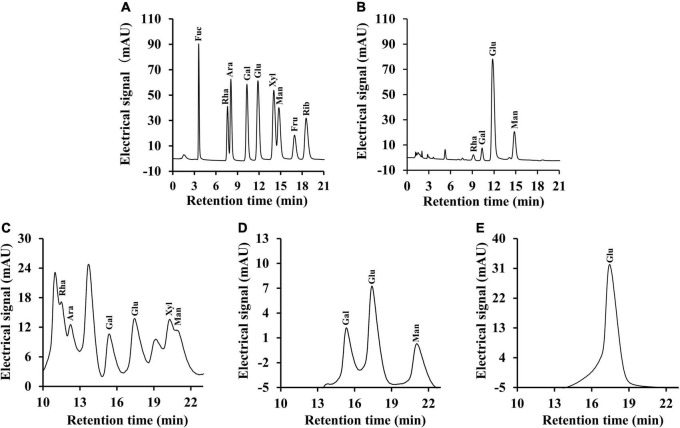
Ion chromatography profiles of the monosaccharide standards, CSFP, CSFP1-β, and CSFP2-α. **(A,C)** The monosaccharide standards, **(B)** CSFP, **(D)** CSFP1-β, and **(E)** CSFP2-α. Fuc, Ara, Rha, Gal, Glc, Xyl, Man, Fru, and Rib represent fucose, arabinose, rhamnose, galactose, glucose, xylose, mannose, fructose, and ribose, respectively.

It is well-known that polysaccharides are formed by a series of monosaccharides linked by glycosidic bonds. On the basis of monosaccharide composition, polysaccharides can be divided into homopolysaccharides and heteropolysaccharides (42). For heteropolysaccharides, in addition to the different types and ordering of their monosaccharide units, heteropolysaccharides also have various types and sequences of glycosidic bonds, which also result in structural diversity (43). As shown in Figure 4, the monosaccharide composition analysis indicated that CSFP were mainly composed of glucose, mannose, galactose, and rhamnose in molar ratios of 1.07: 0.38: 0.11: 0.02. CSFP1-β was mainly composed of glucose, galactose, and mannose in molar ratios of 3.92: 3.61: 2.44. Furthermore, CSFP2-α was a key component of glucose. The results showed that glucose was the most abundant of the three polysaccharides, and CSFP purified by DEAE-cellulose-52 and Sephacryl S-400 could not only change its monosaccharide composition, but also affect its molar ratio. Furthermore, to a certain extent, the monosaccharide composition of polysaccharides affects the biological activity of polysaccharides (44). Liu et al. (45) found that the active substance that played an antioxidant role in the *Agrocybe cylindracea* may be attributed to the presence of Glc and Gal. Hen et al. (46) found that the antioxidant effect of *P. eryngii* may be due to the presence of Man, Rha, and GalA.

In general, the *Mw* distribution of polysaccharides determines its many physico-chemical properties and biological activity (47). Therefore, the *Mw* values of CSFP and its purified components (CSFP1-β, CSFP2-α) were determined by HPGPC in this study. As shown in Table 2, the *Mw* of CSFP was 1.948 × 10^4^ Da. After DEAE-cellulose-52 and Sephacryl S-400 purification, all HPGPC chromatograms of the CSFP1-β and CSFP2-α exhibited two or three peaks, indicating that they were heteropolysaccharides. Results showed that three peaks of CSFP1-β were present at 5.16 × 10^4^ Da (30.62%), 2.462 × 10^4^ Da (38.58%), and 7.086 × 10^3^ Da (30.81%), respectively. In addition, two peaks of CSFP2-α were found at 4.029 × 10^6^ Da (87.12%) and 1.579 × 10^3^ Da (12.88%), respectively. Results showed that the *Mw* values of purified components (CSFP1-β, CSFP2-α) were higher than that of the pectic polysaccharides in okra (*Abelmoschus esculentus*) (48). In addition, polysaccharides with high *Mw* may have better biological activities. Studies have shown that the *Mw* of *Lentinan* is roughly 500 kDa and *Lentinus edodes* polysaccharide is a common, well-established medicinal fungal polysaccharide that has been used as a pharmaceutical agent (49, 50). A polysaccharide-like substance, also called sizofiran, is produced by Schizophyllum, which has a *Mw* of 100–200 kDa (51). Studies have shown that low-*Mw* polysaccharides can penetrate immune cells and stimulate. The superior activity of high-*Mw* polysaccharides may be due to a better binding affinity for carbohydrate receptors of immune cells (52). All in all, the role of *Mw* in the biological activity of polysaccharides is very important.

**TABLE 2:** Changes in molecular weight (*Mw*) and molar ratio of monosaccharide compositions of CSFP, CSFP1-β, and CSFP2-α.

Sample	CSFP	CSFP1-ß	CSFP2-a
Peak 1 *Mw* (Da)	1.948 × 10^4^	5.16 × 10^4^	4.029 × 10^6^
Percentage ratio (%)	100	30.62	87.12
Peak 2 *Mw* (Da)		2.462 × 10^4^	1.579 × 10^3^
Percentage ratio (%)		38.58	12.88
Peak 3 *Mw* (Da)		7.086 × 10^3^	
Percentage ratio (%)		30.81	
**Monosaccharide composition and mole ratio**			
Glucose	1.07	3.92	1
Mannose	0.38	2.44	
Galactose	0.11	3.61	
Rhamnose	0.02		

#### Rheological Characterization

**FIGURE 5:**
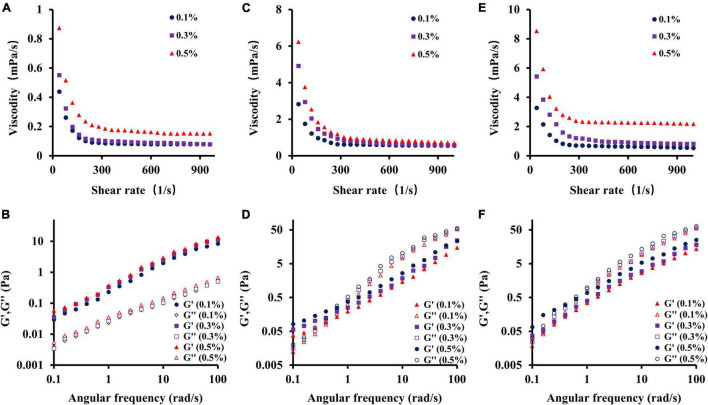
Dependence of apparent viscosity on the shear rate and plots of storage modulus *G’* and loss modulus *G”* against frequency for CSFP, CSFP1-β, and CSFP2-α. **(A,B)** Rheological characteristic diagram of CSFP, **(C,D)** Rheological characteristic diagram of CSFP1-β, and **(E,F)** Rheological characteristic diagram of CSFP2-α.

The rheological properties of natural polysaccharides are related to their antioxidant activities. Owing to their excellent viscosity, they are now widely used in industry to make gelling agents, thickeners, and emulsifiers (44, 53). As shown in Figures 5A,C,E, the apparent viscosity of different samples (CSFP, CSFP1-β, and CSFP2-α) was dose-dependent. This characteristic may be related to the mutual stacking and overlapping of polysaccharide molecules and the increase in the degree of polymerization (54). In addition, the study also found that the apparent viscosity of different samples at different concentrations decreased with the increase in shear rate, and showed the shear-thinning behavior of a Newtonian fluid in the range of high shear rate. The author speculates that the shear-thinning behavior of polysaccharides may be related to the unwinding of molecular chains in solution (55, 56). Moreover, compared with CSFP, the apparent viscosity of purified components (CSFP1-β and CSFP2-α) increased significantly at the same concentration, which may be related to their *Mw* and polydispersity (57). Dynamic rheology techniques can be used to detect the solid or liquid properties of polysaccharides (58). As shown in Figures 5B,D,F. At 25°C, with the increase of angular frequency of CSFP, CSFP1-β, and CSFP2-α, the storage modulus (*G*’) and loss modulus (*G”*) also increase. The change in this characteristic may be related to the increase in polymer chain complexity and the number of connections in the sample between the areas of high concentration (59). In addition, *G*’ and *G”* represent elastic and viscous properties, respectively. When the loss modulus (*G”*) of the sample is greater than the storage modulus (*G*’), it indicates that the sample exhibits liquid-like behavior; otherwise, the sample exhibits solid-like behavior (60). When the concentrations of different sample solutions were 0.1, 0.3, and 0.5%, respectively, *G*’ and *G*” values of CSFP suggested no cross-linking, while cross-formation occurred and the gel formation and elastic properties of CSFP1-β and CSFP2-α increased with increasing concentrations. Hu et al. (61) extracted polysaccharide (DTMP) from “deer tripe mushroom” and studied its rheological and gel properties. The results indicated that the DTMP solution showed shear-thinning behavior (pseudoplasticity), and its pseudoplasticity was more obvious at a concentration of 2%. In addition, DTMP also showed a gel-like behavior (*G*’ > *G*”), and the strength of gel increased with the increase of concentration (from 2 to 10%). In addition, Wang et al. (62) prepared alkali-extracted polysaccharide (HEAEP-0.5) from the water-insoluble residue of *H. erinaceus*. It was found that HEAEP-0.5 was a high-viscosity polysaccharide (its intrinsic viscosity was 1.43 L/g). It exhibited strong shear-thinning behavior and could form weak gels as the concentration increased. Polysaccharides from *Clitocybe squamulosa* have significant rheological properties. This study can provide a theoretical basis for its application in the field of the food industry.

### Bioactivity of CSFP, CSFP1-β, and CSFP2-α

#### Antioxidant Activities

**FIGURE 6:**
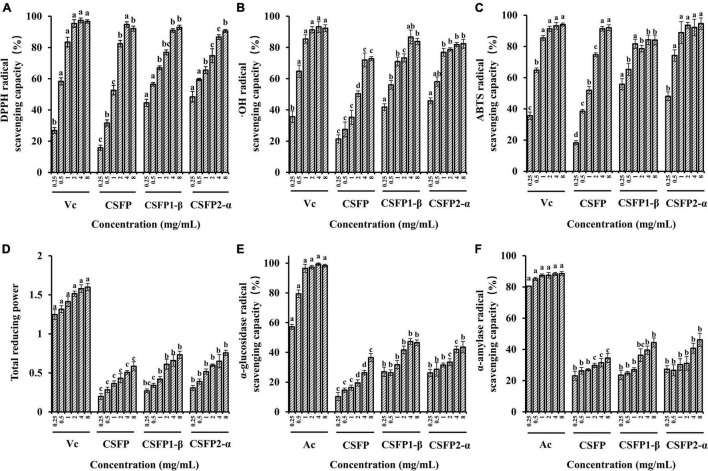
Antioxidant activity and inhibitory enzyme activity of CSFP, CSFP1-β, and CSFP2-α. **(A)** DPPH radical scavenging capacity, **(B)** ⋅OH radical scavenging capacity, **(C)** ABTS radical scavenging capacity, **(D)** total reducing power, **(E)** α-glucosidase inhibition capacity, and **(F)** α-amylase inhibition capacity. Data are expressed as the mean ± standard deviation values (*n* = 3), and different lowercase letters denote statistical significance (*p* < 0.05).

Previous studies have shown that the CSFP has good antioxidant activity (16); however, the antioxidant activity of purified components of CSFP has not been studied. Therefore, this experiment will explore and compare the antioxidant activities of CSFP, CSFP1-β, and CSFP2-α. As shown in Figures 6A–D, compared with Vc, each of CSFP, CSFP1-β, and CSFP2-α showed significant antioxidant activity, and the antioxidant activity of purified components (CSFP1-β and CSFP2-α) was significantly higher than that of CSFP. The IC_50_ values of the DPPH radical scavenging capacity of CSFP, CSFP1-β, and CSFP2-α were 0.828, 0.364, and 0.303 mg/ml, respectively. The IC_50_ values of ⋅OH radical scavenging capacity were 1.763, 0.346, and 0.239 mg/ml, respectively. The IC_50_ values of ABTS radical scavenging capacity were 0.819, 0.106, and 0.203 mg/ml, respectively. Furthermore, three different samples also showed a certain potential for reduction. Research shows that polysaccharides from mushrooms, such as *Volvariella Volvacea*, *Agaricus biporous*, *L. edodes*, and *Pleurotus ostreatus*, exhibited significant antioxidant properties relevant to their health-protecting functions (63–65). Antioxidant heteroglycan (PS, *Mw* ∼ 1.98 × 10^5^ Da) obtained from the aqueous extract of an edible mushroom *Termitomyces clypeatus* (R. Heim) (66) showed ferrous ion chelating ability, superoxide radical scavenging activities, and high reducing power with EC 50 values of 475, 180, and 260 μg/ml, respectively. In addition, polysaccharides rich in uronic acid often have stronger biological effects. Luo et al. (67) confirmed that acidic polysaccharides have more significant antioxidant activity through *in vitro* antioxidant tests on the purified components of ginseng polysaccharides. It was speculated that the antioxidant activity of natural functional polysaccharides was also closely related to their chemical properties and *Mw* distribution (30, 68). Therefore, the polysaccharide component of *C. squamulosa* can be used as a natural antioxidant and has the potential for application in the functional food industry.

#### Enzymatic Inhibitory Activities

Diabetes is another major chronic disease that threatens people’s health. Most hypoglycemic methods on the market include insulin injection and western medicine to control blood sugar concentrations (acarbose, metformin hydrochloride, glimepiride, etc.). The search for new treatments can also reveal new ways to treat diabetes. Studies have shown that the inhibition of α-glucosidase release is one of the main strategies used to combat metabolic symptoms associated with type 2 diabetes and hyperglycemia (69). As shown in Figures 6E,F, CSFP, CSFP1-β, and CSFP2-α have significantly inhibited (*in vitro*) the action of α-glucosidase. Indeed, The IC_50_ values of inhibitory α-glucosidase activity of CSFP, CSFP1-β, and CSFP2-α were 5.20, 8.68, and 22.68 mg/ml, respectively. Compared with Ac, CSFP, CSFP1-β, and CSFP2-α had relatively weak inhibitory effects on α-glucosidase. Moreover, inhibition of α-amylase activity also plays a crucial role in diabetes patients. The IC_50_ values of inhibitory α-amylase activity of CSFP, CSFP1-β, and CSFP2-α were 17.01, 16.85, and 20.92 mg/ml, respectively. Compared with Ac, CSFP, CSFP1-β, and CSFP2-α showed moderate α-amylase inhibitory activity. Studies have shown that the hypoglycemic activity of mushroom polysaccharides is related to its high uridine acid, high degree of esterification, and high *Mw* (28, 70). In addition, the study also reported the hypoglycemic activity of a variety of polysaccharides found in edible fungi. For example, Zhu et al. (71) used the polysaccharide purified from *Ganoderma lucidum* to orally feed diabetic mice for 4 weeks. The results showed that *G. lucidum* polysaccharides decreased fasting blood glucose levels and improved endothelium-dependent aortic relaxation. Chen et al. (72) obtained a heteropolysaccharide from *Grifola frondosa*, and experiments have shown that it could significantly increase glucose uptake, thereby reducing insulin resistance in HepG2 cells and improving glucose levels and glucose tolerance in type 2 diabetic mice. Polysaccharides of edible fungi show good competitiveness in hypoglycemic activity and have the potential for further development.

#### Binding Capacities

**FIGURE 7:**
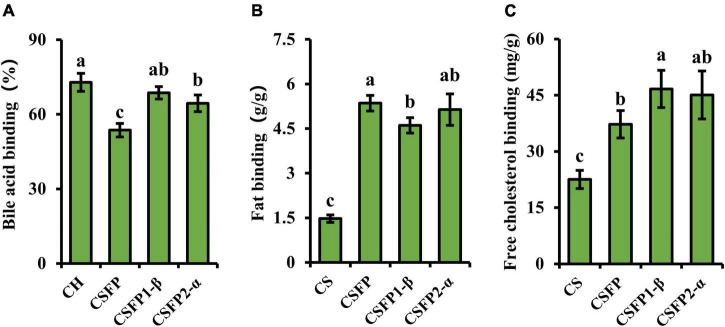
Binding ability of CSFP, CSFP1-β, and CSFP2-α *in vitro*. **(A)** Bile acid, **(B)** Fat, and **(C)** Free cholesterol. Data are expressed as the mean ± standard deviation (*n* = 3) values, and different lowercase letters denote statistical significance (*p* < 0.05).

Excessive intake of bile acids, free cholesterol, and fat often leads to problems such as diabetes, cardiovascular disease, obesity, and so on (44, 73). CSFP can exert good effects on reducing blood lipid and cholesterol content (16). Therefore, this experiment will evaluate and compare the binding ability of purified components of CSFP to fat, cholesterol, and bile acids *in vitro*. As illustrated in Figure 7, the bile acid and cholesterol binding capacities of CSFP were 53.64% and 37.24 mg/g, respectively. After purification, the ability to bind bile acid salt and cholesterol of CSFP1-β and CSFP2-α *in vitro* was significantly improved (*p* < 0.05) to 68.62% and 46.66 mg/g, and 64.43% and 45.05 mg/g, respectively. In addition, although the fat-binding capacity of CSFP1-β and CSFP2-α decreased slightly, they remained significantly higher than that of the positive control (*p* < 0.05). Research showed that the binding ability *in vitro* may be related to the degree of esterification and *Mw* distribution of natural polysaccharides (28, 44). Fu et al. (74) extracted and purified a water-soluble polysaccharide from *Acanthopanax senticosus*. In alloxan-induced mice, oral *A. Senticosus* polysaccharide can reduce the levels of total cholesterol, triglycerides, and low-density lipoprotein cholesterol in mice. In conclusion, all results show that CSFP, CSFP1-β, and CSFP2-α have the potential for application in the prevention of high cholesterol and hyperlipidemia.

#### Antibacterial Activity

**FIGURE 8:**
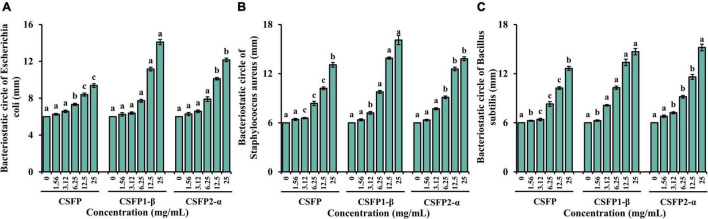
Bacteriostatic test results of CSFP, CSFP1-β, and CSFP2-α. **(A)** *Escherichia coli*, **(B)** *Staphylococcus aureus*, and **(C)** *Bacillus subtilis*. Data are expressed as the mean ± standard deviation values (*n* = 3), and different lowercase letters denote statistical significance (*p* < 0.05).

Nowadays, because many harmful bacteria have developed resistance to antibiotics, it has become even more necessary to control bacterial infections. Therefore, it is necessary to find a new natural pollution-free material for research (75). In this study, the bacteriostatic potential of CSFP, CSFP1-β, and CSFP2-α *in vitro* was evaluated. As shown in Figure 8, within the test concentration range, CSFP, CSFP1-β, and CSFP2-α showed different degrees of antibacterial activity against the three tested strains (*E. coli*, *S. aureus*, and *B. subtilis*). The inhibition zones of CSFP, CSFP1-β, and CSFP2-α for *E. coli* were in the range of 6.36–9.52, 6.24–14.09, and 6.29–12.16 mm, respectively. Studies have shown that *E. coli* is a Gram-negative bacterium, which can cause gastrointestinal or urethral infection in animals under certain conditions. Furthermore, at the same concentration, CSFP1-β has the most significant inhibitory effect on *E. coli*. Polysaccharides from *Periploca laevigata* root barks also showed a good inhibitory effect on *E. coli* (76), which was consistent with the results of this study. Meanwhile, CSFP, CSFP1-β, and CSFP2-α also showed moderate antibacterial activity against *S. aureus* and *B. subtilis*. The antibacterial diameters were (6.41–13.08 mm, 6.36–16.11 mm, and 6.33–13.82 mm) and (6.25–12.65 mm, 6.27–14.68 mm, and 6.80–15.20 mm), respectively. Previous studies have shown that *Cyclocarya paliurus* polysaccharides also show moderate antibacterial activity against *E. coli*, *S. aureus*, and *B. subtilis*. When the concentration of polysaccharide solution was 1 mg/ml, the diameter of the antibacterial ring was 6.54, 6.57, and 6.93 mm, respectively (77). Manna et al. (78) synthesized nanoparticles using a *Lentinus squarrosulus* hetero-polysaccharide and successfully demonstrated its use against *E. coli* and other bacteria. The nanoparticles were better than normal particles in inhibiting bacteria and viruses. Mushroom polysaccharides shielded mice against *Salmonella* lipopolysaccharide-induced septic shock (79). *Auricularia auricula-judae* crude polysaccharides were active against *E. coli* and *S. aureus* (80). A sulfated polysaccharide from oyster mushrooms showed antibacterial activity against food-borne *E. coli* and *S. aureus* (81). The bacteriostatic effect of polysaccharides has been confirmed by many studies, but its related mechanism warrants further studies to provide theoretical support for practical industrial production.

#### Effects of CSFP on RAW264.7 Cells

**FIGURE 9:**
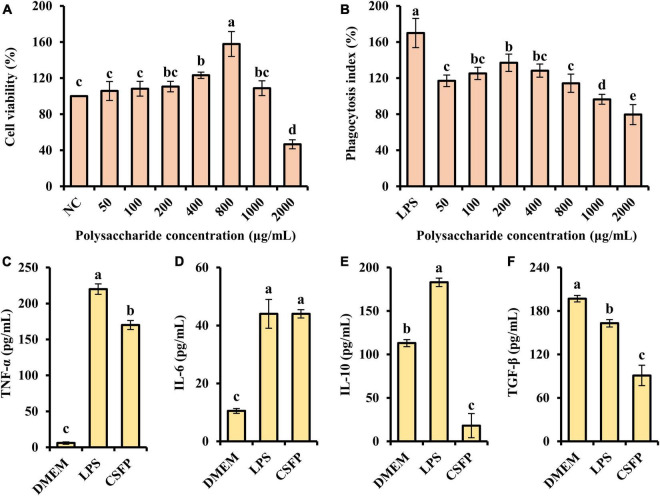
Effect of CSFP on the growth of RAW264.7 cell *in vitro*. **(A)** Cell viability, **(B)** Phagocytosis index, **(C–F)** Secretion of cytokines TNF-α, IL-6, IL-10, and TGF-β. Data are expressed as the mean ± standard deviation values (*n* = 3), and different lowercase letters denote statistical significance (*p* < 0.05).

Macrophages are key immune cells for host defense, and stimulating the activity and phagocytosis of macrophages is an important way of enhancing immune function. The MTT assay and the neutral red assay were used to determine the effect of CSFP on the proliferation of RAW264.7 cells. As shown in Figures 9A,B, within the tested concentration range (50–2,000 μg/ml), CSFP could promote RAW264.7 increment in a dose-dependent manner. When the concentration of CSFP solutions was 800 and 200 μg/ml, respectively, the cell viability and phagocytosis index of macrophages reached their maxima. In addition, the effect of CSFP on the phagocytosis index of RAW264.7 was lower than that of LPS. Research finds that *Glehniae radix* polysaccharide promoted the proliferation of RAW264.7 cells, which suggested that *Glehniae radix* polysaccharide may exhibit potential anti-inflammatory, anti-tumor, and immunoregulation activities (82) in a manner consistent with our study. Therefore, taking the phagocytosis index as the reference, 200 μg/ml of CSFP was selected to treat RAW264.7 macrophages in further studies. RAW264.7 cells were incubated in a high-glucose medium containing 200 μg/ml of CSFP for 12 h and then stimulated with LPS for 4 h. As shown in Figures 9C–F, except for TGF-β, in the determination of TNF-α, IL-4, and IL-10, the LPS group was significantly higher than the DMEM group (*p* < 0.05). In addition, compared with the LPS group, CSFP had a significant effect on the content of cytokines in RAW264.7 cell supernatant (except in the case of IL-6). Furthermore, *Pleurotus nebrodensis* polysaccharides enhanced immunity and inflammatory responses by activating macrophages (83). A previous study indicated the mechanism of macrophage activation induced by a novel polysaccharide (PLCM) extracted from the culture broth of *Cordyceps militaris*. The results showed that PLCM could enhance the immunostimulatory activity of RAW264.7 macrophages, including the release of toxic molecules (NO and SOD), release of cytokine tumor necrosis factor (TNF-α), and the phagocytosis of macrophages (84). The results showed that CSFP could alter the concentrations of inflammatory and anti-inflammatory factors in RAW264.7 cells, thus playing a beneficial role in immune regulation.

## Conclusion

In this study, two new polysaccharides (CSFP1-β and CSFP2-α) were isolated and purified from *C. squamulosa*. The results show that CSFP1-β was mainly composed of glucose, galactose, and mannose, while the main component of CSFP2-α was glucose, and the *Mw* distribution of both was uneven. In addition, purified components (CSFP1-β and CSFP2-α) exhibited good antioxidant activity *in vitro* and have the potential to control post-prandial hyperglycemia and reduce the level of lipids in serum. The study also found that CSFP, CSFP1-β, and CSFP2-α demonstrated a good bacteriostatic effect and showed a linear increasing concentration gradient relationship. CSFP could also significantly promote the proliferation of RAW264.7 cells. Therefore, CSFP, CSFP1-β, and CSFP2-α were expected to become functional food to promote body health for further development and future utilization.

## Data Availability Statement

The original contributions presented in this study are included in the article/supplementary material, further inquiries can be directed to the corresponding authors.

## Author Contributions

DG: conceptualization, methodology, and writing — original draft. JL: software development. LX: investigation and resources. YC: formal analysis and visualization. CF: resources and formal analysis. JM: conceptualization and methodology. MC: resources and data curation. XG: supervision and reviewing and editing of the manuscript. All authors contributed to the article and approved the submitted version.

## Conflict of Interest

The authors declare that the research was conducted in the absence of any commercial or financial relationships that could be construed as a potential conflict of interest.

## Publisher’s Note

All claims expressed in this article are solely those of the authors and do not necessarily represent those of their affiliated organizations, or those of the publisher, the editors and the reviewers. Any product that may be evaluated in this article, or claim that may be made by its manufacturer, is not guaranteed or endorsed by the publisher.
